# Supplementary material for: Examining Bayesian network modeling in identification of dangerous driving behavior
Source: PLoS One. 2021 Aug 13;16(8):e0252484. doi: 10.1371/journal.pone.0252484 (PMC8363010; doi:10.1371/journal.pone.0252484)
Supplement: Attachment — Submitted filename: renamed_b426e.pdf [file pone.0252484.s001.pdf]

Examining Bayesian network modeling in identification of dangerous driving behavior

Manuscript Number PONE-D-21-00035

Reviewers' Comments,

Owing to the rise in the popularity of automobiles over the last century, road accidents have become one of the leading causes of death in many countries around the world. The driver behaviors, such as speeding, drunk driving, and using a mobile phone while driving, are the major factors which lead to inattention of drivers. In addition to avoiding dangerous driving practices yourself, you must learn to identify unsafe behavior in other drivers. When you recognize the risk posed by such drivers, you can act pre-emptively to mitigate that risk by changing your speed, yielding the right-of-way or altering your position on the road. Hence, this manuscript seems an interesting piece of work, and the work sounds fine and good quality. The subject is interesting and emerging. However, I do suggest several important changes to address some weaknesses.

1. Authors are encouraged to add literature review section or related work, such as dangerous driving behavior, dangerous driving behavior identification methods, and so on. What's more, what are the authors' main contributions for the readers?

2. The literature review appears not completely updated. Some latest results on dangerous driving behavior, Bayesian approach and K-means algorithm can be found in:

Zhang, Q., Ge, Y., Qu, W., Zhang, K., Sun, X. (2018). The traffic climate in China: The mediating effect of traffic safety climate between personality and dangerous driving behavior. *Accident Analysis & Prevention*, *113*, 213-223.

Guo, Y., Li, Z., Liu, P., Wu, Y. (2019). Modeling correlation and heterogeneity in crash rates by collision types using full Bayesian random parameters multivariate Tobit model. *Accident Analysis & Prevention*, *128*, 164-174.

Guo, L., Zhou, J. B., Dong, S., Zhang, S. C. (2018). Analysis of urban road traffic accidents based on improved K-means algorithm. *China J. Highway Trans*, *31*(4), 270-279.

Dong, S., Zhang, M., Li, Z. (2020). Risk analysis of vehicle rear-end collisions at intersections. *Journal of advanced transportation*, *2020,* 1-11.

3. In the United States, the most common dangerous driving behaviors which result in collisions are: (a) speeding, (b) driving under the influence of alcohol, (c) distracted driving (including cell phone use and driving while fatigued), (d) reckless or aggressive driving (including tailgating, unsafe lane changes, running stop signs

and failure to yield right-of-way), but in figure 2.13, why the authors only divide the driver's driving style into three types? In addition, how do the authors classify the dangerous driving behaviors? What is the detailed evidence for the classification? How is it different from other literatures? Please double check.

4. The authors employed Bayesian network to construct a vehicle-based traffic crash risk model. Please give more explains and detailed steps for the Bayesian network approach. It is suggested that the authors added the detailed process of the Bayesian network approach.

5. How to prevent these dangerous driving behaviors after the identification?
