# Supplementary material for: Examining Bayesian network modeling in identification of dangerous driving behavior
Source: PLoS One. 2021 Aug 13;16(8):e0252484. doi: 10.1371/journal.pone.0252484 (PMC8363010; doi:10.1371/journal.pone.0252484)
Supplement: Attachment — Submitted filename: PONE-D-21-00035_R2_reviewer.pdf [file pone.0252484.s003.pdf]

Examining Bayesian network modeling in identification of dangerous driving behavior
PONE-D-21-00035_R1

Second Round

May 6, 2021
Reviewer #1

I appreciate the author's extensive revision work. All the comments have been addressed, the topic of this manuscript falls within the scope of *PLOS ONE*, the paper has improved significantly. I recommend it for publication.

Congratulations.

Thank you.

Your reviewer
