# Supplementary material for: Examining Bayesian network modeling in identification of dangerous driving behavior
Source: PLoS One. 2021 Aug 13;16(8):e0252484. doi: 10.1371/journal.pone.0252484 (PMC8363010; doi:10.1371/journal.pone.0252484)
Supplement: Attachment — Submitted filename: Response to Reviewers.docx [file pone.0252484.s002.docx]

Dear editors and reviewers

Thank you for your letter and the reviewers’ comments concerning our manuscript entitled “Examining Bayesian network modeling in identification of dangerous driving behavior”. Those comments are valuable and very helpful. We have read through comments carefully and have made corrections. Based on the instructions provided in your letter, we uploaded the file of the revised manuscript. The responses to the reviewer's comments are presented following.

We would love to thank you for allowing us to resubmit a revised copy of the manuscript and we highly appreciate your time and consideration.

Sincerely.

Leyi Cheng
Corresponding author:
Name: Yichuan Peng
E-mail: [yichuanpeng@tongji.edu.cn](mailto:yichuanpeng@tongji.edu.cn)

Journal Requirements:

1.Please ensure that your manuscript meets PLOS ONE's style requirements, including those for file naming.

Response: Thank you very much for your reminder. We have modified the format of the article as required.

2.We suggest you thoroughly copyedit your manuscript for language usage, spelling, and grammar.

Response: We apologize for the language problems in the original manuscript. The language presentation was improved with careful review.

3.Funding information should not appear in the Acknowledgments section or other areas of your manuscript.

Response: Thank you very much for your reminder. We have deleted the funding information that appeared in the article.

4.Please amend either the abstract on the online submission form (via Edit Submission) or the abstract in the manuscript so that they are identical.

Response: Thank you very much for your reminder. We have amended the abstract on the online submission form.

5.Please ensure that you refer to Figure 2.2, 2.6, 2.7 and 4.4in your text as, if accepted, production will need this reference to link the reader to the figure.

Response: We are grateful for the suggestion. To be more clear and in accordance with the reviewer concerns, we have referred to each figure in our text.

6. We note you have included a table to which you do not refer in the text of your manuscript. Please ensure that you refer to Table 3.2 in your text; if accepted, production will need this reference to link the reader to the Table.

Response: We are grateful for the suggestion. To be more clear and in accordance with the reviewer concerns, we have referred to Table 3.2 in our text.

Reviewer #1

a. Figures should be started from Figure 1.

Response: We are sorry for the format error and we have corrected the name of tables and figures according to journal requirements.

b. the solution of the figures should be improve and high quality figures are preferred.

Response: Thank you for your suggestion. As suggested by reviewer, we deleted the unimportant pictures in the article, thereby simplifying the narrative of the article and at the same time, we have supplemented the conclusions of some pictures.

c. there are too much content in section 2.

Response: We are extremely grateful to reviewer for pointing out this problem. We have adjusted the structure of the article. In the second part of the article, only the data sources and data processing methods are introduced, and the results are displayed in the fourth part of the article. It is convenient for readers to understand and grasp the overall content of the article.

d. the paper should be reorganized as INTRODUCTION, DATA PREPARATION, METHODS, RESULTS, DISCUSSION, CONCLUSION.

Response: Thank you for your suggestion. As suggested by reviewer, we have re-adjusted the structure of the article and added the suggested content to the manuscript which make the structure of the article clearer and easier for readers to understand.

e. several Bayesian related studies could be acknowledged.

Response: We are grateful for the suggestion. As suggested by the reviewer, we have read the article about Bayesian research and added more details about Bayesian research in the first part and the third part of the article.

Reviewer #2

1. Authors are encouraged to add literature review section or related work, such as dangerous driving behavior, dangerous driving behavior identification methods, and so on. What’s more, what are the authors’ main contributions for the readers?

Response: We agree with the comment and have supplemented literature review section. As for authors’ main contributions, our reply is as follows: we developed 8 indicators to comprehensively identify dangerous driving behaviors. Compared with previous studies, this paper considered more types of dangerous driving behaviors, which can ensure the safety of vehicle operation better. And this paper proposes to use Bayesian network to build a vehicle operation risk assessment model which can not only predict the risk but can analyze the correlation between various factors in depth as well.

2. The literature review appears not completely updated.

Response: We deeply appreciate the reviewer’s suggestion. According to the reviewer’s comment, we have supplemented the main methods and development process of Bayesian theory in the method part of the article and supplemented the background content in the introduction part of the article. And the cited documents have also been added and updated.

3. In the United States, the most common dangerous driving behaviors which result in collisions are: (a) speeding, (b) driving under the influence of alcohol, (c) distracted driving (including cell phone use and driving while fatigued), (d) reckless or aggressive driving (including tailgating, unsafe lane changes, running stop signs and failure to yield right-of-way), but in figure 2.13, why the authors only divide the driver's driving style into three types? In addition, how do the authors classify the dangerous driving behaviors? What is the detailed evidence for the classification? How is it different from other literatures? Please double check.

Response: Our deepest gratitude goes to you for your careful work. the discussion regarding this question is presented following: by reading relevant literature on dangerous driving behaviors in the past, we summarized 8 dangerous driving behaviors based on vehicle trajectory data which can ensure the safety of vehicle operation better. As for the classification of driving style, we use the K-means++ method to classify the driving style of the driver, and use the elbow method to determine the number of classifications. From **Fig 4,** we can see that when the number of clusters is less than 3, the change in SSE is large, and when the number of clusters is greater than 3, the change in SSE tends to be flat. Therefore, we determine that the number of classifications is 3.

4. The authors employed Bayesian network to construct a vehicle-based traffic crash risk model. Please give more explains and detailed steps for the Bayesian network approach. It is suggested that the authors added the detailed process of the Bayesian network approach.

Response: We deeply appreciate the reviewer’s suggestion. According to the reviewer’s comment, we have supplemented the main research process and results of Bayesian theory in the method part, and supplemented the details of the MCMC algorithm and counting algorithm used in the article. The specific steps and results of the algorithm are displayed in the results section.

5. How to prevent these dangerous driving behaviors after the identification?

Response: We are grateful for the suggestion. To be more clear and in accordance with the reviewer concerns, we have added a brief description as follows: once the risk level reaches a higher level, the model infers the most likely driving behavior that causes the danger based on the posterior probability, and reminds the driver to respond.
